# Impact of improved small-scale livestock farming on human nutrition

**DOI:** 10.1038/s41598-020-80387-x

**Published:** 2021-01-08

**Authors:** Md. Emran Hossain, Md. Ahasanul Hoque, Emanuele Giorgi, Guillaume Fournié, Goutam Buddha Das, Joerg Henning

**Affiliations:** 1 Chattogram Veterinary and Animal Sciences University (CVASU), Chattogram, Bangladesh; 2 Lancaster Medical School, Lancaster University, Lancaster, UK; 3 Department of Pathobiology and Population Sciences, Royal Veterinary College, London, UK; 4 School of Veterinary Science, University of Queensland, Gatton, QLD 4343 Australia

**Keywords:** Health care, Nutrition, Epidemiology, Sustainability

## Abstract

Micronutrient deficiencies and stunting rates are high in many low-income countries. Increasing and diversifying food intake are often challenging for small-scale farmers in lowland areas as flooding often results in crop losses and drowning of livestock. A cluster-randomised controlled trial was conducted over 12-months in Bangladesh, involving 150 small-scale duck rearing households, including 50 control, and 50 households each in two intervention arms. Interventions focussing on improving duck health and duck nutrition were applied on a village level. Data analysis focussed on assessing the impact of interventions on duck mortality, sales and consumption, and on dietary diversity of household members. Improved duck rearing increased the consumption and the sales of ducks. Household selling more ducks were more likely to purchase and consume milk products, contributing to an improved households’ dietary diversity. Our results suggest that improving duck rearing can provide a suitable and sustainable alternative to maintain and improve dietary diversity of households in flood-prone areas.

## Introduction

In 2015, the United Nations Member States proposed 17 Sustainable Development Goals (SDGs), which require urgent actions to achieve a better and sustainable future for everyone^1^. These goals are interconnected, requiring strategies that go hand-in-hand to reduce poverty, inequality, climate and environmental degradation and ensuring prosperity, peace and justice^2^. The food, agriculture and livestock sector could offer key solutions for sustainable development, as it is central for hunger and poverty eradication in developing countries, such as Bangladesh^3^. Malnutrition in Bangladesh is among the highest in the world, with 36% of children under 5 stunted^4^, and almost half of the population of children under 5 being anaemic^5^. This malnutrition usually encompasses a combination of an inadequate intake of energy, micronutrients and protein^6^.

Studies have shown that plant-based complementary foods by themselves are insufficient to meet the needs for certain micronutrients^7^. Nonetheless, animal source foods (ASF) are an excellent energy-dense source of high-quality and readily digested protein with an array of micronutrients^8^ and can contribute greatly to overcome the deficiencies in diet quantity and quality in children^9^. Studies among Cambodian children aged 12–59 months and among Indonesian children aged 0–59 months showed that consuming a diverse diet with ASF was significantly associated with a reduction in stunting^10,11^.

Among the ASF, poultry meat and eggs in particular can provide more bioavailable vitamins, minerals, protein and fat than plant-based foods^12^. Poultry raising is popular among rural households in developing countries due to its ease of management and slaughtering and little input requirements^13^. In addition, poultry can be quickly sold when money is needed, for instance to purchase other food items and as women generally own and raise village poultry, their sales are a potential source of income and empowerment^14^.

In a review of animal production interventions, Leroy et al*.*^15^ highlighted that scientific evidence is needed to demonstrate that the nutritional status of humans can be improved through interventions promoting animal production. However, research exploring the impact of livestock production-based interventions on human nutrition has mainly been observational, and evidence is limited to show a direct link between improved livestock production and human health and nutrition. For example, data collected in questionnaire survey in Tanzania indicated that numbers of chickens owned was correlated with a reported minimum number of food groups consumed in households, although a causal relationship could not be shown with study design used^16^.

Also, while some studies have focussed on free-ranging chickens^17^, duck production provides more advantages compared village chicken production: (1) duck eggs are larger and provide more protein and (2) anecdotic evidence indicates that some households prefer to raise ducks for cultural or religious reasons. In addition, natural disasters such as flooding can exacerbate food insecurity and worsen dietary diversity among subsistence farmers due to losses of crops^18,19^ or due to the drowning of chickens in floodwaters and are often associated with long-term malnutrition in rural communities^20,21^. The implementation of long-term malnutrition prevention programmes after floods in flood-prone areas is recommended, but their implementation is difficult^22^. Duck rearing can provide here a suitable alternative. As ducks can swim in rising water levels and as they are more resistant to diseases than chickens, ducks are better adapted to environmental challenges^23^. Thus, duck production could provide an important strategy to sustain adequate ASF production to meet nutritional needs of rural households exposed to frequent flood events.

The one-health framework provides an opportunity to explore questions at the intersections of human, animal, and environmental health by using approaches that are used in these individual scientific domains^24^. It aims to use expertise across these disciplines to develop an enhanced understanding of a range of health impacts and to develop integrated approaches to complex questions relating to human health in conjunction with animal and environmental health^25^.

Thus, under this one-health framework, we aimed to assess the impact of interventions targeting duck rearing practices on nutritional outcomes of rural households in flood-prone areas of Bangladesh. We hypothesized that simple and sustainable interventions relating to improving duck health and nutrition would (1) enhance duck production, and (2) that the resulting increased availability of duck meat and eggs would increase their consumption and sales, and (3) improve dietary diversity of household members. The results would provide a holistic trajectory that improved duck production can provide a sustainable agricultural intervention to reduce malnutrition in an environment under severe pressure through climate change.

## Results

### Impact of improved duck rearing interventions on duck sales and duck consumption

**Figure 1:**
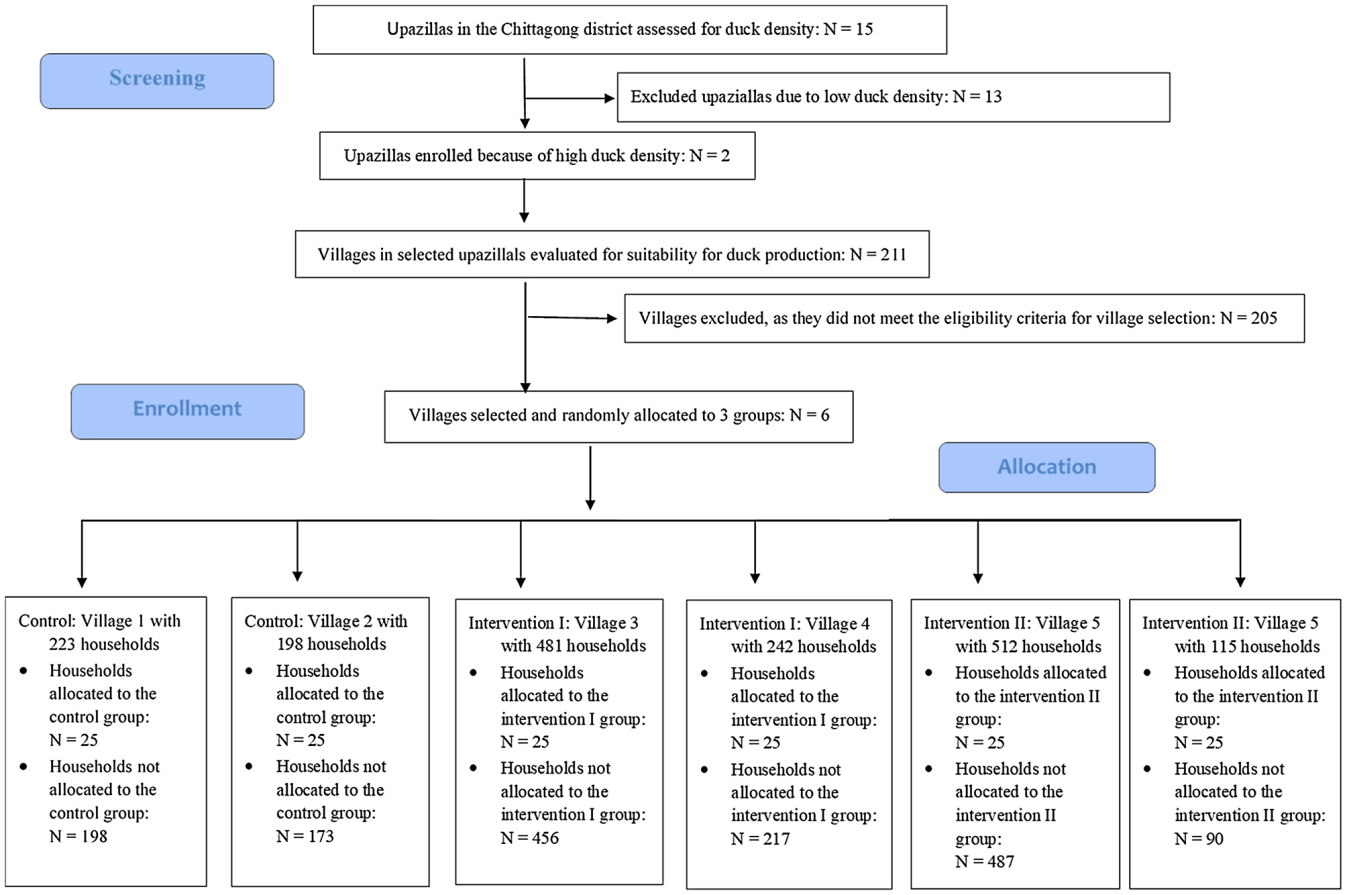
Flowchart of design of the cluster-randomized controlled trial.

Between July 2016 to June 2017, 150 duck-rearing households across six villages were allocated to into three treatment groups: (1) a Control group where households received 15 ducklings, training in duck rearing and a bamboo basket to protect ducklings, (2) an Intervention I group where households received, in addition to the supplies and training as in the Control group, deworming and vaccination of ducks, and (3) an Intervention II where households received, in addition to the supplies and training as in the Control group and the deworming and vaccination of ducks as in Intervention I, also duck feed over a period of 4 months (Fig. 1).

All Intervention I and II and 66.0% (N = 33) of Control households were Muslim (N = 17 of Control households were Hindu). As, to be eligible, households should have some experience in duck rearing, it was not surprising that some recruited households already owned some ducks (N = 57, 38.0%) (Control: N = 18, 31.6%; Intervention I: N = 23, 40.3%; Intervention II: N = 16, 28.1%). However, the mean flock size of these “pre-existing” duck flocks at the beginning of the study was only 4.8 ducks for Control, 5.8 and 3.5 for Intervention I and II households and did not differ between these groups (p = 0.417, Table 1). Some baseline characteristics (household size, wealth index) were similar for households in the treatment (Table 1), although the Household Dietary Diversity Score (HDDS) and experience in duck rearing in years was slightly lower in Intervention I and slightly higher in Intervention II group compared to the Controls. Therefore to account for differences between households’ characteristics at baseline, the difference of the HDDS at each month to the baseline month was compared between the three groups. The focus of the analysis was only on the ducks supplied to households as (a) all of the supplied ducks were of the same age allowing comparisons between groups, (b) the interventions targeted the supplied ducks and not on the “pre-existing” ducks, and (c) the supplied ducks accounted for most of the flock size.

**Table 1:** Enrolment characteristics of duck raising households by treatment groups.

Baseline characteristics	Control (N = 50) Mean (SD)	Intervention I (N = 50) Mean (SD)	Intervention II (N = 50) Mean (SD)	p-value
Household size	6.22 (1.80)	6.02 (1.90)	6.20 (2.05)	0.661
Annual income (Bangladeshi Taka)	148,835 (143,674)	166,992 (120,022)	186,878 (218,267)	0.038
Wealth index	− 0.57 (0.65)	− 0.60 (1.70)	− 0.57 (0.68)	0.186
Experience in duck rearing (years)	12.04 (6.34)	9.70 (8.86)	12.26 (9.48)	0.038
Flock size of existing ducks*	4.78 (3.95)	5.81 (13.81)	3.52 (1.85)	0.417
Household dietary diversity score	6.32 (0.89)	5.22 (1.00)	7.06 (1.18)	0.001

*Number of households with existing ducks: control N = 18, intervention I N = 23, intervention II N = 16.

**Figure 2:**
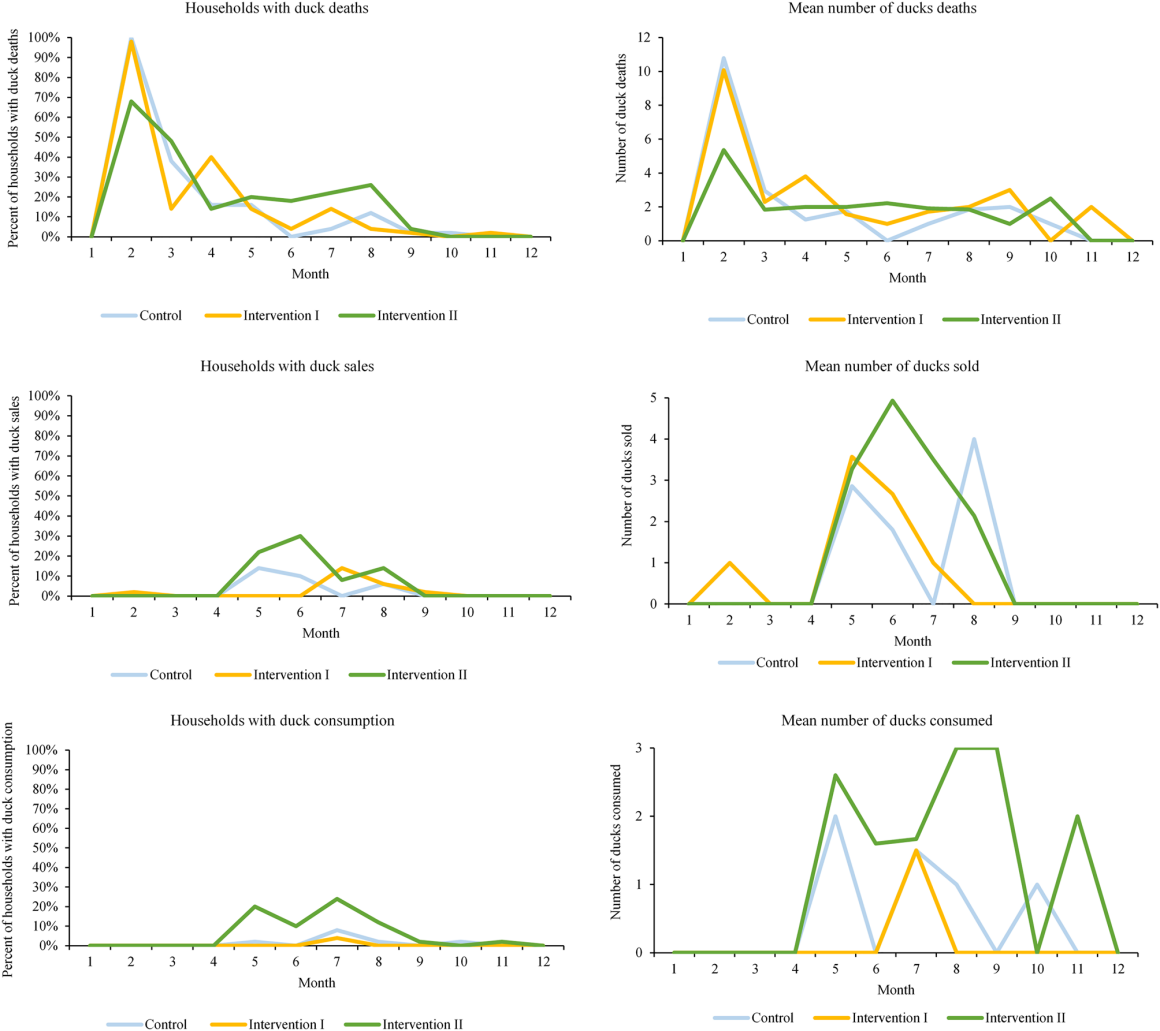
Percentage of households experiencing deaths, selling and consuming ducks by treatment groups and mean number of duck deaths, duck sales and ducks consumed by treatment group over the 12-month observation period.

Raw data on the percent of households with sales, consumption and mortalities of ducks are shown in Fig. 2. The proportion of households with duck mortalities was the highest in the first four months. Overall, a similar proportion of households experienced duck mortalities in Control and Intervention I households, but the proportion of households experiencing duck mortalities and mean number of duck deaths were considerably lower in Intervention II households (Fig. 2). In the second four months of the study period, the mean proportion of households consuming and selling ducks was 17% and 19% for Intervention II, 1% and 5% for Intervention I and 3% and 8% for Control households, respectively (Fig. 2). As ducks can only be eaten or sold when they have a sufficient weight at about 5 months, the consumption or sales of ducks peaked at this age. Among Intervention II households selling ducks, a larger number of ducks were sold per month (up to five ducks) and sales continued over serval months. Similarly, in households consuming ducks, consumption was more consistent over a period of 5 months and conducted in larger numbers (up to three ducks) compared to Intervention I and in Control households.

Furthermore, at the end of the study period, 98% of the Intervention II, but only 54% of Intervention I and 40% of Control households had still supplied ducks in their flocks (Supplementary Fig. S1). Across all households, a mean of 4.0 supplied ducks were still present in Intervention II households at the end of the study period, compared to a mean of 1.8 and 1.4 ducks in Intervention I and in Control households, respectively (Supplementary Fig. S1).

Using the continuous time-to-event models for the two competing risks of death and sale or consumption, we were able to estimate the predicted monthly probability of ducks dying, being sold or consumed under the three study arms. Monthly probability of ducks death peaked at 15% in Control, 11% in Intervention I, but was significantly lower in Intervention II villages (up to 5%) (Supplementary Fig. S2), with considerable variation between villages. The estimated monthly probability of consumption or sales of ducks was twice as high for Intervention II villages as Control and Intervention I villages (Supplementary Fig. S2).

From these models, we also estimated median time until death and until sale and consumption for the three study arms. The 95% confidence interval for time until death was 2–6 months in Control and Intervention I villages, but with 5–22 months much wider for Intervention II villages (Table 2).

**Table 2:** Estimated median time until death of ducks and until consumption or sale of ducks by treatment groups.

Treatment group per village	Median time until death (months) (95% CI)	Median time until sale or consumption (months) (95% CI)
Control—village 1	3.73 (2.45, 4.77)	11.82 (6.20, 17.60)
Control—village 2	3.44 (2.83, 3.95)	14.58 (9.04, 20.30)
Intervention I—village 3	3.71 (2.37, 4.75)	12.50 (6.68, 18.36)
Intervention I—village 4	5.40 (4.81, 6.03)	15.18 (9.37, 20.98)
Intervention II—village 5	7.53 (4.82, 10.30)	12.40 (7.69, 17.07)
Intervention II—village 6	13.88 (6.64, 21.70)	13.14 (8.66, 17.73)

### Changes to nutritional outcomes of households members

**Figure 3:**
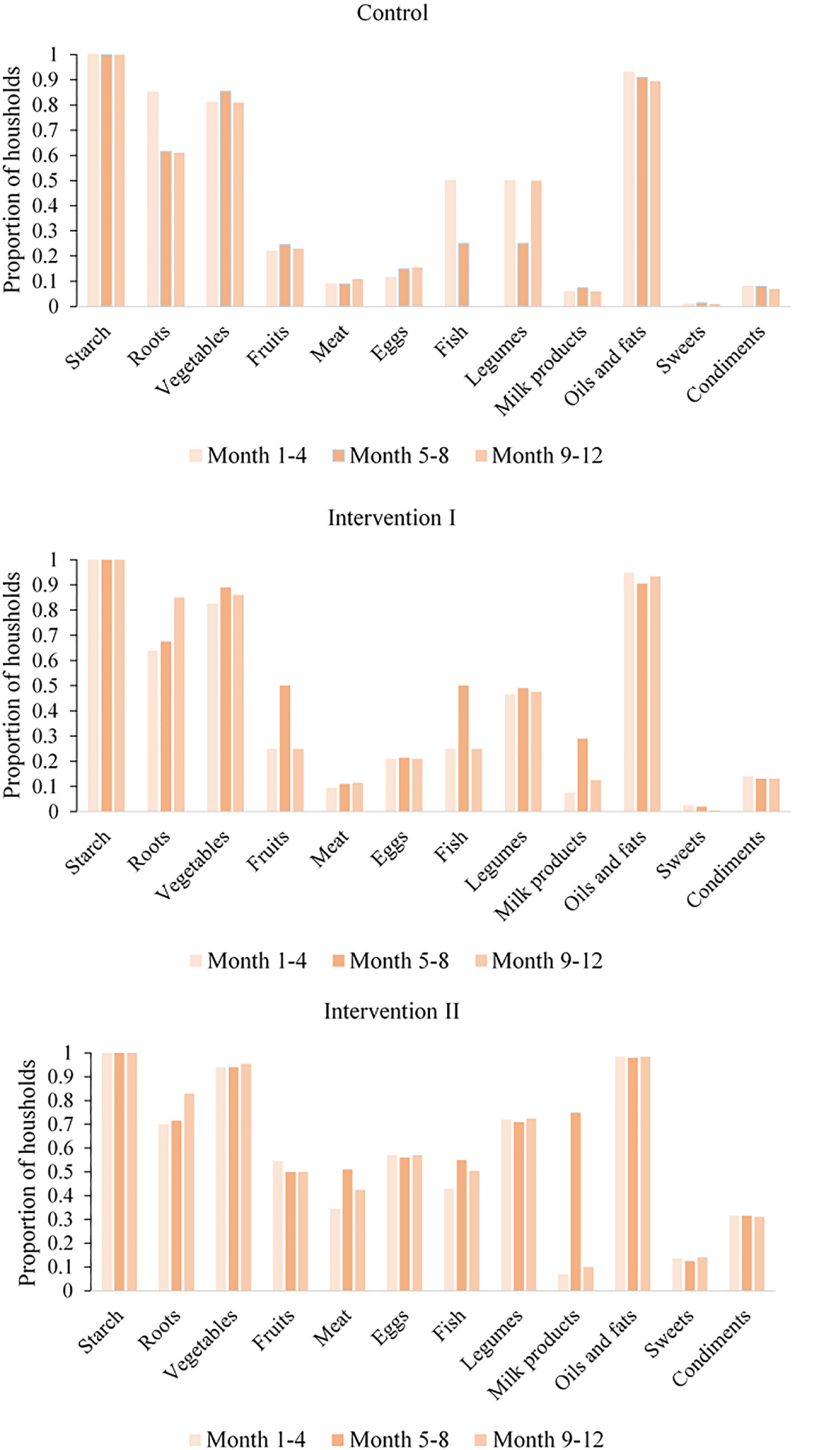
Proportion of households in each treatment group that consumed food items in four-monthly intervals over the 12-month study period.

The observed monthly HDDS over the study period is shown in Supplementary Fig. S3 indicating an increase in the HDDS for the two intervention groups. Exploring the food groups that contributed to the HDDS over time, an increase in the consumption of duck meat in Intervention II group from 34% of households in the first four months of the study to 51% of households in the second four-month period of the study was noticeable (Fig. 3). In Control households the proportion of households consuming meat was constant at 9%, and in Intervention I households it increased from 10 to 12% between the two four-month periods. In addition, in the second four months of the study, when sales of ducks were high, there was an increase in the proportion of households purchasing and consuming milk products in Intervention II (from 7 to 75%) and in Intervention I (from 8 to 29%), but only marginally in Control households (from 6 to 8%). There was no substantial change in the proportion of households consuming eggs between the study periods for the three study arms. Ducks do start laying eggs at about 5–6 months and supplied ducks at this age were consumed or sold rather than kept for further egg production.

**Figure 4:**
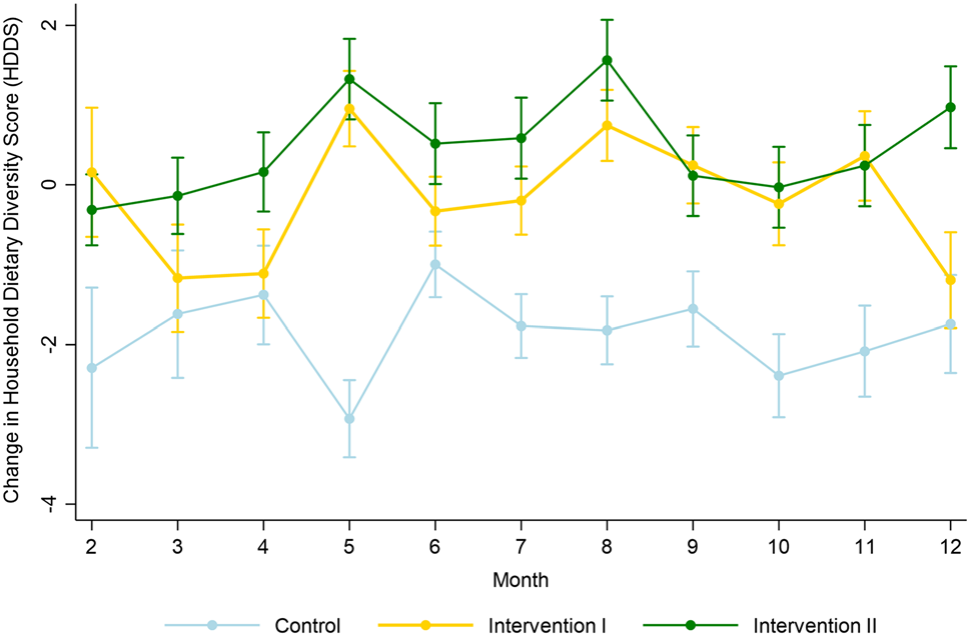
Estimated change in the Households Dietary Diversity Scores (HDDS) (with 95% confidence interval) by treatment groups over a 12-month observation period.

As the HDDS differed slightly between the three groups at the beginning of the trial, the focus of the analysis was on the change of the HDDS over time while adjusting for time-varying variables, i.e. the estimated monthly probability of ducks deaths and sale or consumption of ducks and a monthly health score for each household capturing adverse health events experienced within each household in each month (Supplementary Fig. S4). The proportion of households with adverse health events increased over time, and peaked from January to May for all three groups. There was a strong interaction between the month of the study and the intervention groups (Supplementary Table S1). Overall, interventions markedly improved the HDDS, with the largest HDDS improvement at five months relative to the baseline month with a HDDS change of 1.0 (95% CI 0.5, 1.4, *P* < 0.0001) and 1.32 (95% CI 0.8, 1.8, *P* < 0.0001) for Interventions I and II, respectively (Fig. 4).

## Discussion

The implemented interventions promoted duck survival, and, subsequently, increased dietary diversity in members of households that were rearing these ducks. To our knowledge, only one cluster-randomized trial involving livestock has been conducted, where a range of items were provided to participants, including vegetable and fruit seedlings, gardening tools and in some cases chickens, and then dietary intake of mothers and their empowerment was measured^26^. However, interventions in this trial were complex and the specific effect of livestock rearing on dietary diversity was not measured. Thus, this is the first cluster randomized trial that focussed directly on the effect of improved livestock rearing on HDDS.

The HDDS used in this study reflects the ability of a household to access a variety of foods^27^ and is therefore associated with the household’s food security^28^. The HDDS also provides an indication of household economic access to food, which is either produced by the household or purchased through household resources^27^. Households with interventions that prevented the occurrence of duck diseases through vaccinations and in particular supported the growth of ducklings by the provision of duck starter feed (Intervention II), increased their HDDS. Likely explanations of the mechanism helping households to increase their HDDS are increased consumption of duck meat and/or the households increased purchase power due to increased duck sales. In the months with increased duck sales, additional income was generated, that allowed households to purchase food items that expanded their dietary diversity. This included the purchase and consumption of more milk products in the months with high duck sales. Although the sex of ducks consumed or sold was not recorded, households are more likely to consume (or sell) male ducks, while female ducks are kept for producing new ducklings.

We are confident that the increased dietary diversity identified in this study was a result of the improved duck productivity and that no serious confounders had been present. Five of our study villages were not involved in any research or development projects that could have provided resources to households to improve their dietary diversity. However, one study village, recruited in the Intervention II arm, was part of the National Agricultural Technology Program (NATP) which was implemented by Government of Bangladesh between 2014 and 2017. In this program, cattle and supporting health services (e.g. vaccination, deworming) were provided to 20 out of 512 households in Tinchoudia—none of these 20 households were included in our study.

The provision of poultry to rural households for the establishment of flocks is a common strategy implemented by NGOs in developing countries, but such an approach is likely to fail, if no veterinary support and advice is provided to prevent poultry mortalities. For free-ranging poultry, deaths are the highest in the first few months after hatching, mainly due to predation and an inadequate supply with a protein rich diet. These constraints were addressed in our study^29,30^. Thus, to develop successful and sustainable livestock-based interventions aiming to improve the nutritional status of people, expertise from veterinarians, agronomists, agriculture economists as well as doctors, public health researchers and human nutritionists need to be considered. Our cross-disciplinary research highlighted that agriculture actions such as deworming, vaccination and supply of feed are required to increase productivity of ducks (which is similar to the use of fertilizers for crops) and can subsequently result in the improvement of nutritional outcomes in humans. Our research was conducted in low-lying areas with limited agricultural opportunities due to flooding and it highlighted that duck rearing can provide an alternative where other crop production or livestock production such as chicken raising are not possible due to rising water levels. Thus, the outcomes of our research will be also of relevance to policy-makers managing human nutrition programmes, especially in lowland areas where extreme weather events can have a severe impact on the production of plant-based foods.

It could be argued that improvements in duck productivity, consumption of duck meat and increase in HDDS might only reflect farmer’s practices and behaviours during the study period as a direct result of “artificially” introduced interventions. We are confident that this is not the case and that the farmers would not return back to their traditional duck management practices. In fact, all participating farmers were asked at the end of the intervention study if they would adopt the promoted interventions and 100% of farmers in intervention group II and 90% of farmers in intervention group I indicated that they will adopt these interventions with the aim to sell supplementary ducks, rather than just consuming them. Although the primary objective of the present study was to assess the effectiveness of these interventions in improving dietary diversity, the only way to confirm that these interventions will be sustained, would be through a long-term follow-up study.

Unfortunately when new technologies are introduced in developing countries, subsidy levels are initially high, but once funding is withdrawn, such programs often fail^31^. The concept of ‘inclusive innovation' utilizes local assets and involves local knowledge and skills, and thereby provides an approach that transitions toward sustainability^32^. We followed this framework by inviting households with duck rearing experience to participate in our trial, while developing and promoting simple and easily adoptable interventions for this flood-prone environment. Thus, study households had to fulfil some minimum conditions for duck rearing (at least one of year duck raising experience, existence of a duck house and access to waterways). The reason was that we needed to ensure that households had at least some familiarity with duck raising and a suitable environment to reduce the risk of ducklings dying. Importantly, all households (Control and Intervention I and II) received the same training in duck raising providing similar baseline conditions to all households. As the promoted interventions focussed on duck rearing, the external validity of our study results mainly relates to duck producers. Nevertheless, duck production is the most suitable livestock production type in flooded areas, thus our results are applicable to a large number of households in these agro-ecological zones.

Villages represented clusters in the cluster-randomized trial and interventions were applied to all households within a village. Based on our discussions with villagers and village elders, this was the only possible design to “avoid contamination” bias between households and to limit envy about the success of interventions, when different interventions are used within a village.

We used a 24-h recall period to monitor changes in diet diversity^33^. The recall period of 24 h is less subject to recall error, less cumbersome for the household member and also conforms to the recall time period used in many dietary diversity studies^33–35^. However, it has to be considered that seasonal differences in dietary patterns might exist, which could be influenced by food shortages or they can be a result of natural disasters. Furthermore, during religious and seasonal festivals, dietary patterns might also change, but the majority of households in our study were Muslim. We monitored households over a year, but for a more complete assessment of dietary diversity, long-term studies evaluating the impact of the promoted intervention on dietary diversity while considering seasonal factors should be conducted.

Apart from dietary diversity information and data on adverse human health events, no other human health parameters were measured. Household owners were not willing to have blood samples collected to measure health and nutrient related blood parameters at the beginning of the study, but they were willing to do so at the end of the study after a trustful and close relationship had been developed with the researchers. This might be a limitation of our research, although the data on adverse health events that we had captured could be considered as a surrogate for negative “health benefits” that households could have experienced.

Many of the baseline characteristics of households did not differ between the three study arms (household size, flocks size of existing ducks, wealth index), while others did vary (experience in duck rearing, annual income and HDDS). We addressed this issue by comparing the change of the HDDS at each month to the baseline month between the three groups. Reporting bias in the number of ducks dead/sold/consumed could might have occurred in our study, but we tried to limit this bias by requesting household owners to record all mortalities or sales and consumption as soon as they had occurred in a recording book.

Through a One-Health approach, we were able to demonstrate that improving the health status of farmed animals, in this case ducks, can have a direct impact on the dietary diversity, and therefore, on the health of the humans who raise them.

In conclusion, the interdependence between farmed animal and human health are often emphasised in the literature through the One-Health concept, but research on the impact of animal health interventions on human nutrition is extremely rare. Our study is the first to assess the impact of specific improved duck rearing interventions on dietary diversity in humans. The study supports the use of animal-resource food for human nutrition and provides evidence that improved duck rearing is a feasible and sustainable approach to improve dietary diversity. As floods often cause crop losses and malnutrition in low-income and middle-income countries, improved duck rearing practices can provide a suitable alternative to promote micronutrients and protein intake under challenging environmental conditions.

## Methods

### Study design

We conducted a cluster-randomized trial over a period of 12 months in two upazillas (sub-districts) (Anwara and Rangunia) of the district of Chattogram, Bangladesh. These upazillas were selected because of their high duck density. Rangunia upazilla comprised of 46,176 households and a total area of 351.95 km^2^, while Anowara upazilla had 38,008 households and a total area of 164.13 km^2^.

Two villages in Rangunia (Tinchoudia and Lalanagar) and four villages in Anwara (Raypur, Sorenga, Gohera and Dudhkumra) were selected based on the following criteria: (1) access to waterways, which provide a scavenging environment for ducks to obtain feed, (2) minimum distance of 10 km between villages to minimise contamination bias and (3) reasonable assess and moderate costs associated with travel to these villages. The waterways proving scavenging opportunities for ducks represented rivers of Hoar deltas and were prone to flooding, which often result in damage or destruction of crops produced by the households in these villages.

The cluster-randomised controlled trial comprised of three arms—an Intervention I, an Intervention II and a Control arm. Interventions were applied on a village level as it was likely (based on discussion with village elders) that multiple interventions applied to households within a village would result in contamination—i.e. households being exposed to and adopting interventions which were not scheduled for them—or conflict among households allocated to different arms.

### Eligibility

The purposively selected villages were randomly distributed into the three arms, with two villages per arm (Control villages: Gohera and Dudhkumra, Intervention I villages: Sorenga and Lalanagar, Intervention II villages: Raypur and Tinchoudia) (Supplementary Fig. S5).

Within each village, a sampling frame consisting of all households was obtained from the village elders. Households were then visited sequentially based on random numbers generated for each village and assessed for four eligibility criteria: (1) a household member must have had at least one year of experience in duck rearing, (2) a household had to have a duck house, and (3) the household must have access to a water reservoir containing scavenging feeds and (4) the duck managing household member had to provide informed consent to be involved in the study. It was also confirmed that none of the household selected had been involved in previous or current development or research projects. If the criteria were not fulfilled for a given household, the next household on the list was visited. The procedure stopped once 25 households had been recruited in each study village. Thus, 50 households were enrolled in each study arm (Fig. 1).

### Interventions

All households received (a) 15 ducklings, (b) a bamboo basket and (c) training in duck rearing. Both intervention groups received preventive duck health interventions, which included deworming and vaccination. In addition, Intervention II group was provided with duck feed.

Ducklings were day-old unsexed ducks of a similar proportion of four breeds (Khaki Campbell, Indian Runner, Jinding and Pekin) purchased from the Regional Government Duck Breeding Farm, Sonagazi, Feni, Bangladesh. Their average initial weight was 40.0 g. Spiral ring bands were attached to the feet of supplied ducks to distinguish them from potentially existing ducks within the same flock.

The bamboo basket was provided to protect ducklings from predators and to keep them warm in the first weeks after provision to the household. Training in duck rearing was provided to all households over a period of 10 days (1–10 July 2016). A training session lasted four hours and included all aspect of raising ducks, from breeding, hatching, incubation, brooding of ducks, housing, feeding, vaccination and the prevention and control of the main duck diseases. In this training, the nutritional benefits of consuming ASF (duck meat and eggs) were also discussed. Two duck production experts from Chattogram Veterinary and Animal Sciences University (CVASU), Bangladesh, conducted the training. At the end of the training, a question and answer session was conducted and a training manual on duck rearing in Bangla language was provided to all households.

Anthelmintic (Levamisole) were given to the ducklings in the two intervention groups first at the age of 40 days and then repeatedly every 60 days. Duck vaccination was conducted in both Intervention groups. Duck plague and duck cholera vaccines were provided from the Department of Livestock Services, Chattogram, Bangladesh. Duck plague vaccine was given intramuscularly at the age of 30 days and duck cholera vaccine was given subcutaneously at the age of 60 days. Booster vaccinations were provided after 15 days and additional vaccinations were conducted every six months.

For the first four months of the trial, each household in the Intervention II group received commercially produced pellet feed (Diameter = 5 mm; ME = 3000 kcal/kg; CP = 22%; CF =  < 5%; EE =  < 5%; Ca = 0.9%; Pavail = 0.4%; Lysine = 1%; Methionine = 0.4%) at the rate of 1.0 kg per duckling per month.

### Data collection

A baseline questionnaire was used to collect the following data: number of household members, religion practiced in the household, livestock ownership, experience in duck rearing, agricultural land size, approximate annual income, assets owned by the household (to develop a wealth index—see below) and food groups consumed (to develop a household baseline dietary diversity—see below). A follow-up questionnaire was then administered every month to collect information about duck mortalities, the consumption and sale of ducks and eggs, the occurrence of adverse health events among household members within the last month and household dietary diversity in the 24-h period prior to the interview. All interviews were conducted by two co-authors.

### Measurements of dietary diversity

Dietary diversity was recorded at the household level, and included all foods eaten by any member of the household, excluding foods eaten outside the home^27^. Food items were categorized into 12 groups which included cereals (e.g. bread, rice noodles, biscuits, or any other foods made from millet, sorghum, maize, rice, wheat), roots and white tubers (e.g. potatoes, yams, manioc, cassava), vegetables, fruits, duck meat, eggs, fish (fresh or dried fish and other seafood), legumes (e.g. beans, peas, lentils), nuts and seeds, milk and milk products (e.g. cheese, yogurt), oils and fats (e.g. butter, palm oil), sweets (e.g. honey, cookies) and condiments (e.g. coffee, tea, spices, soya sauce). If a household consumed at least one food item under a given food group, the score for this group was 1, and 0 otherwise. The Household Dietary Diversity Score (HDDS) was the sum of the 12 food group scores (i.e. maximum score of 12).

### Statistical analysis

Data at baseline (household size, annual income, experience in duck rearing, flock size of existing ducks and household wealth index, HDDS) were compared between the three groups using the Kruskal Wallis test. The wealth index summarized the ownership of assets by each household at the start of the trial: the presence of a TV, sewing machine, bicycle and radio (binary variables), the number of cattle and chickens. The wealth index was assessed for each household through a factor analysis^36^. Standard methods of performing factor analysis (i.e. those based on a matrix of Pearson’s correlations) assume that variables are continuous and follow a multivariate normal distribution. However, if some variables are dichotomous as in our dataset, a polychoric correlation matrix needed to be developed. Based on this polychoric correlation matrix^37^, the factor with the highest Eigenvalue (which was accounting for 50.0% of the variance) was identified. An orthogonal varimax rotation was then applied and the score for the factor with the highest Eigenvalue (based on the rotated factor loadings) was estimated for each household.

Further data analysis focussed on assessing the impact of the interventions on (1) duck mortality, duck sales and duck consumption and (2) the dietary diversity of households. The death, sale and consumption of ducks were considered as mutually exclusive events. Duck consumption and sales were combined into one category representing a positive or desirable outcome, while mortality represented a negative or adverse outcome. In other words, if a duck died, this duck could not contribute to the cohort of ducks that were sold or consumed. We developed a continuous time-to-event model for the two competing risks of death (*j* = 1) and sale or consumption (*j* = 2). This takes into account that we did not observe the exact time of occurrence of a given event, but that the event occurred between two consecutive visits (i.e. within a given month). More specifically, we assumed that the time-to-event follows a Weibull distribution with village-specific shape and scale parameters. The reason for this is that households from the same village received the same interventions while heterogeneity in village characteristics, such as scavenging conditions (e.g. availability of scavenging feed, access, distribution and quality of water reservoirs), may have affected the mortality, sale and consumption of ducks. Parameters were estimated through a Bayesian inferential framework. We specified informative priors for the parameters that regulate the overall distribution of the mortality, sale and consumption of ducks within the 12-month period of the study. By sampling from the posterior distribution of model parameters, we then estimated the probabilities of “death” and “sales or consumptions” for each of the 12 months. More technical details on the competing risks model, including the derivation of the prior distributions, are provided in the Supplementary Information.

We considered health status as an important confounder as households might modify their diet based on the adverse health events they had experienced. The health status represented the sum of a total of 30 different adverse health events that could have occurred within a month. If a household experienced an adverse health event in a month, it was coded as one, otherwise as zero.

To account for changes in HDDS while also addressing differences between households’ characteristics at baseline, the difference of the HDDS at each month to the baseline month was calculated. We used Multilevel mixed-effects linear regression to compare the change in HDDS between control and intervention households, while accounting for clustering of observations by including village and household ID’s as random effects. Fixed effects included the estimated monthly probability of duck deaths and sale or consumption of ducks at a village level, a variable capturing the monthly health status of household members, the month of observation, the treatment group (Control, Intervention I and Intervention II) and the interaction between month of observation, the treatment group.

The continuous time-to-event model for the two competing risks was fitted using the R software 3.5.1^38^, while all other data analysis was conducted in STATA 15.0 (Stata Statistical Software: Release 14. College Station, TX: StataCorp LP).

### Ethical statement

Institutional approval for conducting interviews was obtained from the University of Queensland, Human Research Ethics Committee (No. 2016000342).We confirm that the randomized clinical trial was performed in accordance with the guidelines and regulations of the University of Queensland, Human Research Ethics Committee. Informed consent was obtained from all participants before the start of the trial.

### Trial Registration

The trial was registered with the Australian New Zealand Clinical Trials Registry (ANZCTR), registration number: ACTRN12618001803280.

## Supplementary Information

Supplementary Information

## Data Availability

The dataset analysed in this study is available from the https://doi.org/10.6084/m9.figshare.11971929.
