# Supplementary material for: Impact of improved small-scale livestock farming on human nutrition
Source: Sci Rep. 2021 Jan 8;11:191. doi: 10.1038/s41598-020-80387-x (PMC7794515; doi:10.1038/s41598-020-80387-x)
Supplement: Supplementary file 1 — Supplementary Information [file 41598_2020_80387_MOESM1_ESM.pdf]

Impact of improved small-scale livestock farming on human nutrition

Md. Emran Hossain, Md. Ahasanul Hoque, Emanuele Giorgi, Guillaume Fournie, Goutam Buddha Das, Joerg Henning

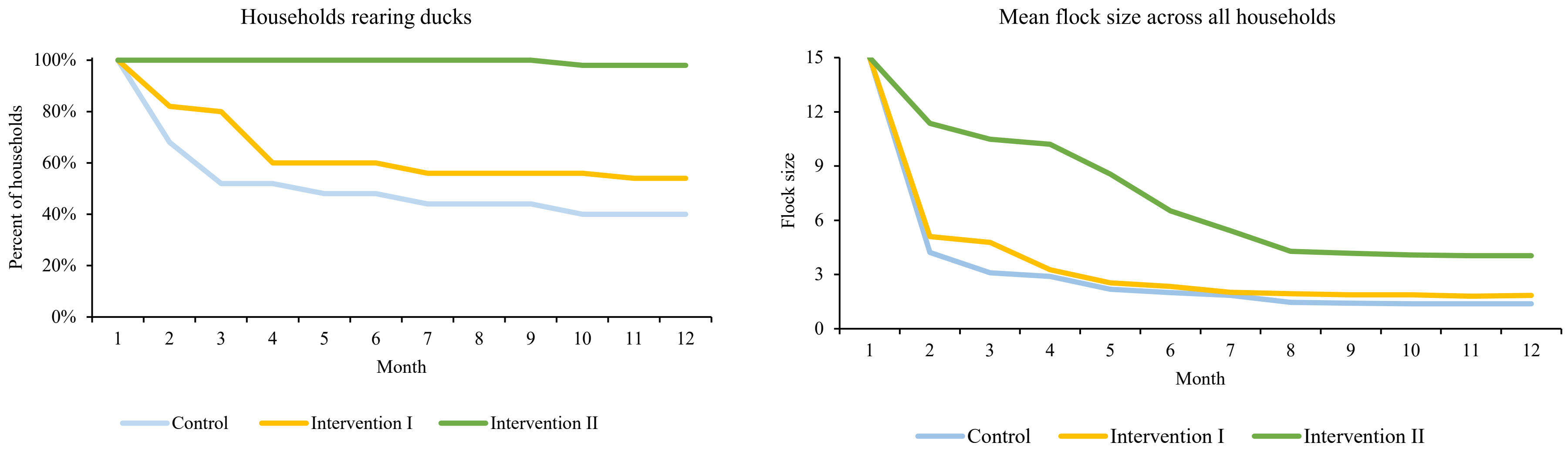

Supplementary Figure S1. Percentage of households rearing ducks by treatment groups and mean flock size across all households by treatment group over a 12-month observation period.

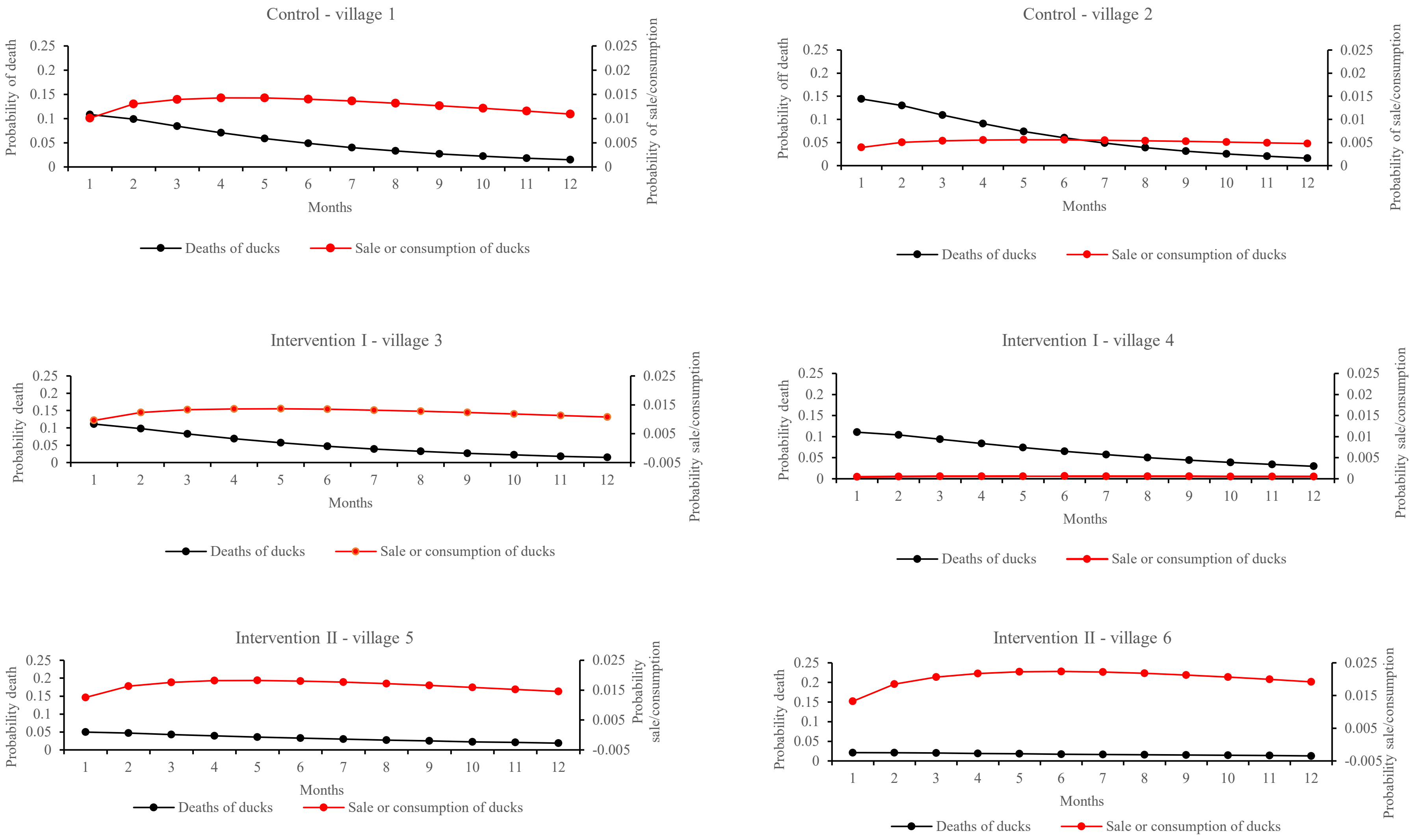

Supplementary Figure S2. Estimated monthly probabilities of death and of sale/consumption of ducks by treatment groups for individual study villages.

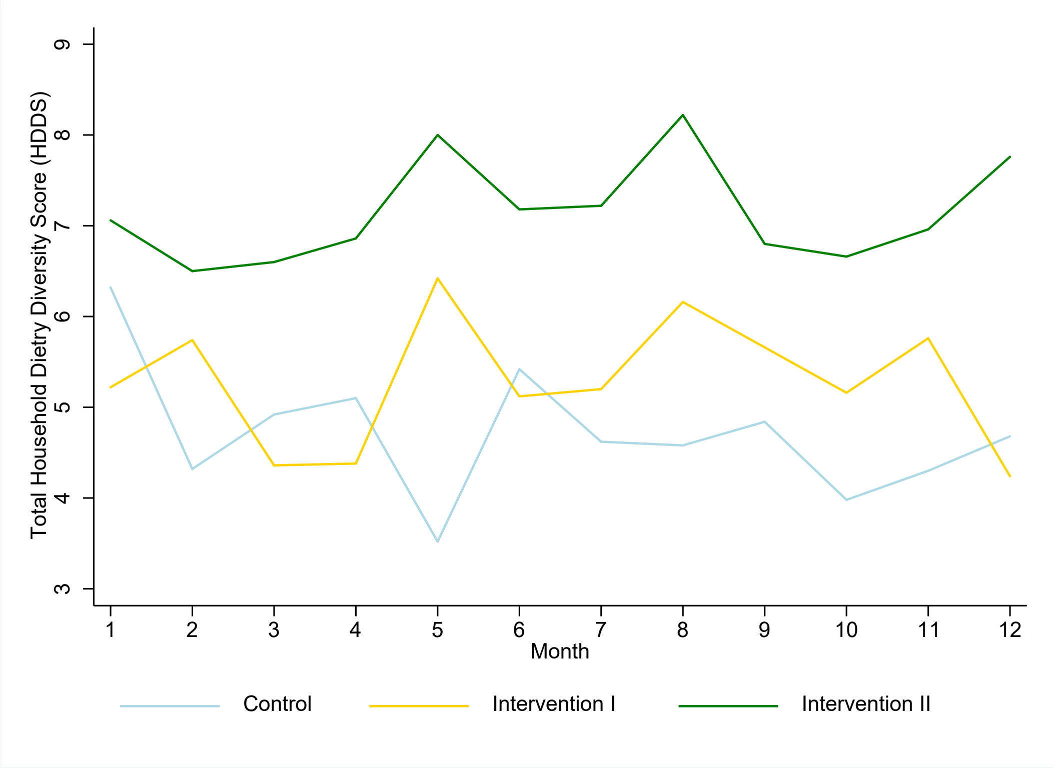

Supplementary Figure S3. Households Dietary Diversity Scores (HDDS) by treatment groups over a 12-month observation period.

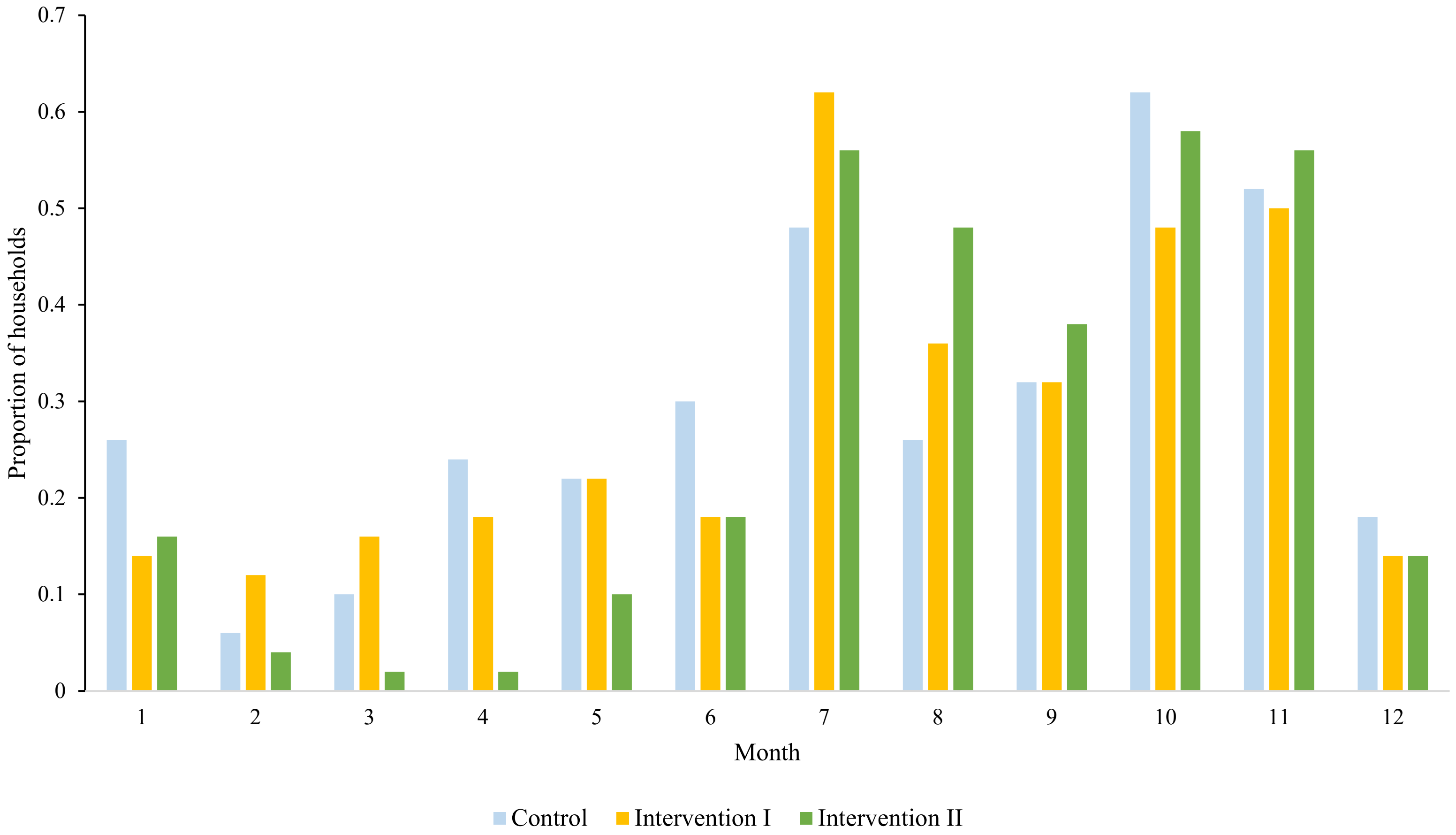

Supplementary Figure S4. Proportion of households with adverse health events (health score) in each treatment group over the 12-month study period. The following adverse health events were monitored within each household: 1. Allergy, 2. Arthritis, 3. Asthma, 4. Cancer, 5. Cold, 6. Constipation, 7. Convulsion, 8. Diabetes, 9. Diarrhoea, 10. Dysentery, 11. Eye Infection, 12. Fever, 13. Gastric problems, 14. Headache, 15. Heart problems, 16. High blood pressure, 16. Leg injury, 17. Jaundice, 18. Kidney infection, 19. Low blood pressure, 20. Mental problems, 21. Paralysis, 22. Pneumonia, 23. Pox, 24. Thalassemia, 25. Tonsillitis, 26. Tuberculosis, 27. Tumour, 28. Typhoid, 29. Vomiting, 30. Weakness.

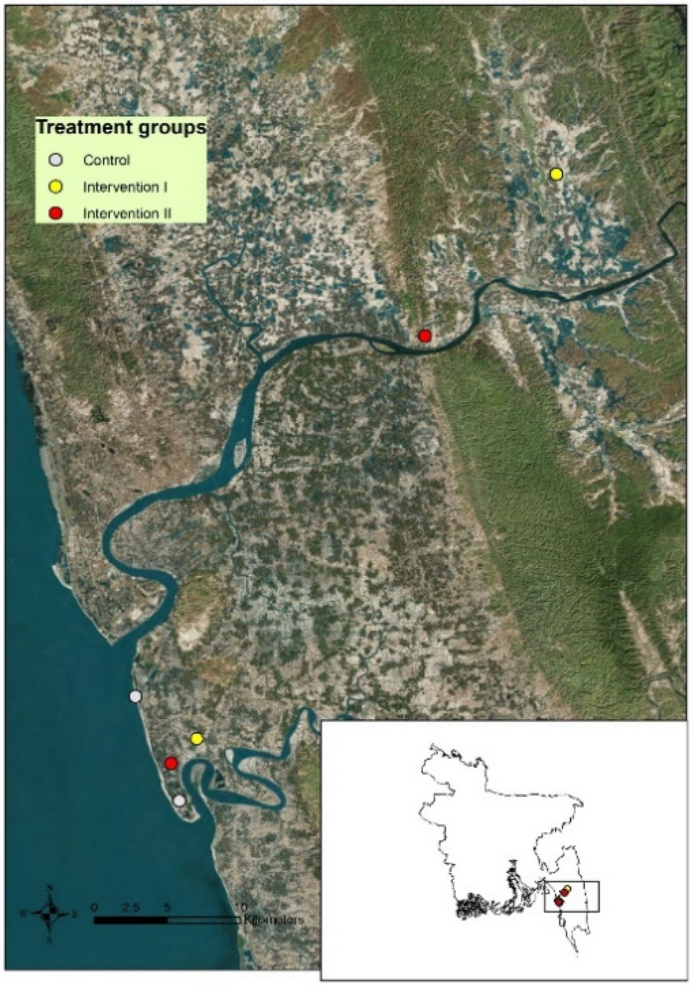

Supplementary Figure S5. Map of study villages located in the district of Chattogram, Bangladesh. The map as was created using ArcMap 10.8 (Esri Inc., https://www.esri.com).

Variables	Coefficient	95% CI lower value	95% CI upper value	p-value
Probability of death	2.28	-11.47	16.03	0.745
Probability of sale and consumption	-42.19	-83.80	-0.58	0.047
Health Score	-0.09	-0.21	0.03	0.127
Control	Reference			
Intervention-I	2.45	1.84	3.06	<0.001
Intervention-II	1.98	0.84	3.12	0.001
Month 2	Reference			
Month 3	0.67	0.22	1.13	0.004
Month 4	0.91	0.31	1.51	0.003
Month 5	-0.64	-1.39	0.12	0.100
Month 6	1.29	0.39	2.20	0.005
Month 7	0.52	-0.50	1.56	0.323
Month 8	0.47	-0.66	1.61	0.423
Month 9	0.74	-0.49	1.98	0.245
Month 10	-0.10	-1.41	1.22	0.884
Month 11	0.21	-1.16	1.59	0.770
Month 12	0.54	-0.89	1.98	0.457
Intervention-I x Month 3	-1.99	-2.57	-1.43	<0.000
Intervention-I x Month 4	-2.18	-2.76	-1.60	<0.000
Intervention-I x Month 5	1.44	0.84	2.02	<0.000
Intervention-I x Month 6	-1.78	-2.38	-1.18	<0.000
Intervention-I x Month 7	-0.88	-1.49	-0.27	<0.005
Intervention-I x Month 8	0.12	-0.50	0.74	0.707
Intervention-I x Month 9	-0.65	-1.27	-0.02	0.042
Intervention-I x Month 10	-0.30	-0.93	0.33	0.354
Intervention-I x Month 11	-0.00	-0.64	0.63	0.993
Intervention-I x Month 12	-1.90	-2.53	-1.27	<0.001
Intervention-II x Month 3	-0.50	-1.11	0.12	0.112
Intervention-II x Month 4	-0.44	-1.15	0.28	0.233
Intervention-II x Month 5	2.28	1.44	3.11	<0.001
Intervention-II x Month 6	-0.46	-1.40	0.48	0.336
Intervention-II x Month 7	0.377	-0.65	1.41	0.474
Intervention-II x Month 8	1.41	0.31	2.51	0.012
Intervention-II x Month 9	-0.31	-1.47	0.85	0.603
Intervention-II x Month 10	0.38	-0.82	1.59	0.536
Intervention-II x Month 11	0.35	-0.89	1.59	0.582
Intervention-II x Month 12	0.73	-.052	1.00	0.251
Constant	-1.87	-3.70	-0.05	0.044

Supplementary Table S1. Final multivariable model for a change in Households Dietary Diversity Scores (HDDS) by treatment groups. The dependent variable in the model is the change in the change in the HDSS. The model was estimated using a multilevel mixed-effects linear regression framework.

Supplementary Methods (page 8-10)

Additional information on the continuous time-to-event model for the two competing risks of death and sale or consumption

Notation

- i = village
- j = type of event: j = 1, "death"; j = 2, "sold or consumed"
- t = month
- Y_{ijt} = random variable of the number of ducks in village i and time t for which event j has occurred between $t-1$ and t.
- C_{it} = random variable of the number of ducks in village i for which neither of the two event of interests has occurred between $t-1$ and t.
- T_{ij} = unobserved random variable of the time of occurrence for event j.

The continuous time-to-event model for competing risks of death and sale/consumption

We develop a continuous time-to-event model for the two competing risks "death" (j = 1) and "sold or consumed" (j = 2). Let $Y_{it} = (Y_{i1t}, Y_{i2t})$ be the random variable of the number of ducks that die (Y_{i1t}) and are sold or consumed (Y_{i2t}) in village i and month t. We assume that the random variable of the time-to-event j, say T_{ij}, follows a Weibull distribution with scale λ_{ij} and shape κ_{ij}, hence

$$P(T_{ij} < t) = 1 - \exp\{-(t/\lambda_{ij})^{\kappa_{ij}}\}.$$

Let C_{it} denote the number of ducks that neither died nor were sold or consumed in village i at month t. We then model the random vector (Y_{it}, C_{it}) as

$$P(Y_{it} = y_{it}, C_{it} = c_{it} | \kappa_{ij}, \lambda_{ij}) = \frac{(\sum_{j=1}^{2} y_{ijt} + c_{it})!}{\prod_{j=1}^{2} y_{ijt}! c_{it}!} \prod_{j=1}^{2} p_{ijt}^{y_{ijt}} \left(1 - \sum_{j=1}^{2} p_{ijt}\right)^{c_{it}} \quad (1)$$

Where

$$\begin{aligned} p_{ijt} &= \pi_{ij} P\left(t-1 < T_{ij} < t \,|\, T_{ij} > t-1, \kappa_{ij}, \lambda_{ij}\right) \\ &= \pi_{ij} \left[\exp\{-[(t-1)/\lambda_{ij}]^{\kappa_{ij}}\} - \exp\{-[t/\lambda_{ij}]^{\kappa_{ij}}\}\right] / \exp\{-[(t-1)/\lambda_{ij}]^{\kappa_{ij}}\} \quad (2) \end{aligned}$$

with the constraint that $\pi_{i1} = 1 - \pi_{i2}$.

We fit the model using Bayesian methods of inference. We specify informative priors for the shape (κ_{ij}) and scale (λ_{ij}) parameters of the Weibull distribution as follows. We define Gaussian priors truncated in 0 such that

$$P(l_{ij} < \lambda_{ij} < L_{ij}, m_{ij} < \kappa_{ij} < M_{ij}) = 0.95$$

where l_{ij}, L_{ij}, m_{ij} and M_{ij} are hyper-parameter values that are chosen so as to give with 95% prior probability: a mortality in ducks over the 12 month period between 40% and 60% for Control villages (i = 1, 2), between 30% and 50% for Intervention I villages (i = 3, 4) and between 20% and 40% for Intervention II villages (i = 5, 6); a sale or consumption of ducks over the 12 month period between 30% and 50% for all villages. For the mixing probabilities π_{ij} we use non-informative uniform priors on the unit interval.

The resulting prior distributions that respect these constraints are shown in Fig. S8.

We sample from the posterior distributions of λ_{ij}, κ_{ij} and π_{ij} using a Metropolis-Hastings algorithm which we implemented in the R software environment (version 3.5.1).

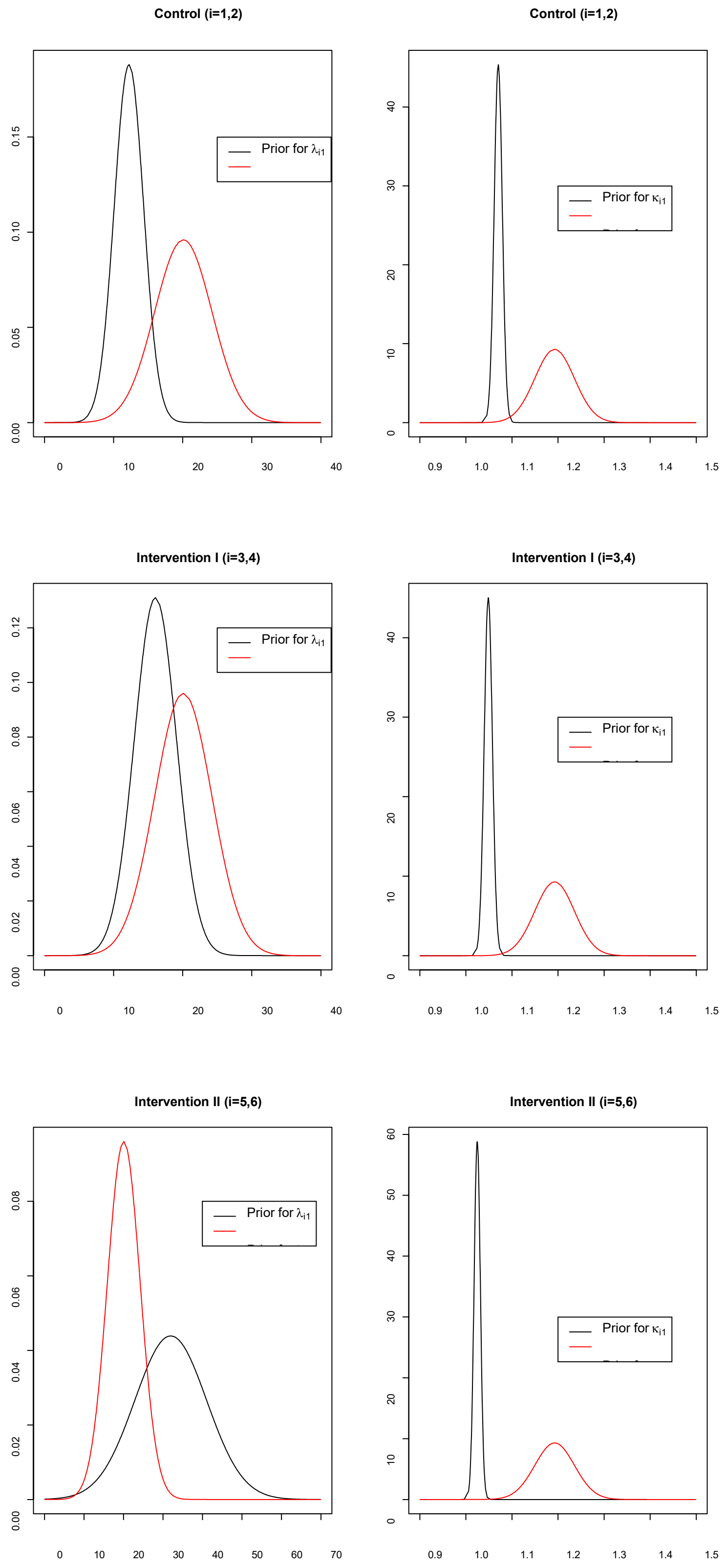

Prior distributions for the scale (λ_{ij}) and shape (κ_{ij}) parameters of the Weibull distributions.
